# National Consensus on Introducing and Delivery of Paediatric Praziquantel for Curative and Preventive Treatment of Schistosomiasis Among Pre-school Children in Tanzania

**DOI:** 10.24248/eahrj.v9i2.843

**Published:** 2025-12-24

**Authors:** Kijakazi Obed Mashoto, Prince Pius Mutalemwa, Corinne Simone Collette, Edward Mberu Kamau, Marie-eve Raguenaud, George Kabona, Mohamed Nyati, Paul Erasto Kazyoba

**Affiliations:** a National Institute for Medical Research, 3 Barack Obama Drive, Dar es Salaam, Tanzania; b Special Programme for Research & Training in Tropical Diseases (TDR), World Health Organization, Geneva, Switzerland; c Neglected Tropical Diseases Control Programme, Ministry of Health, Dodoma, Tanzania

## Abstract

**Background:**

Global and national strategies to control and/or eliminate schistosomiasis focus mainly on MDA for School-Aged Children (SAC) and in some areas, adults. Following growing demand for specific intervention for paediatric schistosomiasis, paediatric praziquantel (arPZQ) formulation has been developed, and it is expected to be on market in 2025.

**Objective:**

The objective of the project was to prepare the health system for the introduction and delivery of paediatric praziquantel to pre-school aged children in Tanzania.

**Methodology:**

This was cross-sectional implementation research which employed qualitative methods including group discussions, consultative meetings, and stakeholder's engagements at different levels.

**Results:**

The engagement of multi-stakeholders from national to community levels led to the identification of implementation gaps and challenges which have the potential to delay the introduction of arPZQ. Through engagement process, stakeholders addressed the gaps and identified potential delivery models relevant to the context of Tanzanian health system.

**Conclusion:**

To ensure no pre-school child is left behind, consensus was reached to target children in the communities and those visiting health facilities for routine healthcare or child health growth monitoring services, and during vitamin A supplementation campaigns. Prior to starting the delivery of arPZQ, capacity of the healthcare facilities to deliver arPZQ should be strengthened to ensure healthcare workers do the right thing.

## BACKGROUND

Schistosomiasis is a neglected tropical disease that affects nearly 220 million people worldwide.^1,2^ It is caused by *Schistosoma* trematodes, transmitted through contact with *Schistosoma* cercariae contaminated water. Depending on the species involved, either urogenital or gastrointestinal tract may be affected. The disease mostly leads to disability rather than death.^2^ Long-term infections are associated with anaemia, growth stunting, impaired cognitive development and work capacity, and later in life, an increased risk of bladder cancer and infertility.^3–5^ Schistosomiasis is widely considered a ‘disease of poverty, being endemic in the tropical and subtropical regions with inadequate water supply and sanitation infrastructure. According to the World Health Organization, over 90% of all *Schistosoma* infections are in sub-Saharan Africa (SSA).^1^

Global and national strategies for control and elimination of schistosomiasis have mainly focused on Mass Drug Administration (MDA) among School-Aged Children (SAC) and in some settings, adults. Recent WHO guidelines on the control and elimination of human schistosomiasis recommend annual Preventive Chemotherapy (PC) with a single dose of praziquantel, administered at ≥75% coverage across all age groups from 2 years and above, including adults, in areas with a prevalence of Schistosoma infection ≥10%.^6^ Although this target may be adequate in moderate and low transmission settings, areas with high prevalence of >50%, require consistently higher levels of therapeutic coverage achieved through multiple rounds of MDA.^7,8^ Thus, two rounds of MDA are recommended annually in regions where prevalence exceeds 50%.^5^ This approach increases the likelihood of reaching individuals who miss the first round, thereby supporting effective control or elimination of the disease. It should be noted that untreated individuals can yield a large number of eggs into the environment, potentially generating a rebound in disease prevalence despite the MDA activities.^9,10^

Official reports indicate that, despite recent progress, Africa is yet to achieve the recommended treatment coverage targets for SAC. As of 2019, approximately 61.8 million SAC and 11.2 million adults received preventive chemotherapy against schistosomiasis in the region, representing 57.1% and 11.9% of those deemed to require treatment for each age group.^11^ However, this intervention did not include children under the age of five years despite the growing body of evidence which indicates that the prevalence of paediatric schistosomiasis is equally high in most endemic areas.^12–14^ A recent study in the Southern highlands of Tanzania reported *Schistosoma mansoni* prevalence among preschool children in Nyasa District Council (DC), Ruvuma region, of 15% and 21% when screened using Kato-Katz technique and point of care circulating cathodic antigens (PoC-CCA) respectively.^15^ This evidence necessitates the need to prepare health systems for the delivery of arpraziquantel (arPZQ) once approved for clinical use. Consequently, this study engaged multi-stakeholders through a participatory approach to identify and address potential health system barriers, and to co-develop national strategies for the introduction and delivery of paediatric praziquantel (pPZQ) in Tanzania.

## METHODS

### Study Design

This study was a cross-sectional implementation research project employing qualitative methods, including consultative technical meetings, stakeholder's consultative workshop, group discussions and community engagement meetings.

### Data Collection

**Consultative technical meeting:** Key institutions responsible for the introduction of new health technologies participated in the consultative technical meeting (Table 1). These included the Tanzania Medicines and Medical Devices Authority (TMDA), Medical Stores Department (MSD), Ministry of Health departments namely, Pharmaceutical Services Unit (PSU), the Department of Preventive Medicines through the Neglected Tropical Diseases Control Programme (NTDCP), and the Department of Maternal, Reproductive and Child Health; as well as the National Institute for Medical Research (NIMR). Participants identified gaps and bottlenecks that could delay the timely introduction and delivery of arPZQ and proposed feasible delivery models suited to Tanzania's health system. Key outputs from this meeting were later presented at the national level consultative workshop.

**TABLE 1:** Stakeholders Engagement and Consultation

Level	Participants	Aim
Community	Ward and village leaders, ward development committee members, village health committee members, community health workers, health care providers, religious leaders, primary school teachers, and other influential people	Create awareness of the burden of schistosomiasis in children ≤5 years.
		Create awareness about the imminent availability of pediatric praziquantel in 2023/2024.
		Seek community opinions and views on delivery models for the delivery of pediatric praziquantel.
Council and Regional	Members of Regional and Council Health Management Teams	Information sharing on the burden of Schistosomiasis infection among children ≤5 years.
		Discuss & consolidate inputs on the delivery models prior to the pilot delivery of pediatric praziquantel in 2023/2024
National	Chief Medical Officer, Director of Preventive Services, Director of Pharmaceutical Services, Director of Maternal, Reproductive and Child Health, Director of Health and Social Welfare Services at the PO-RALG, Manager of NTDCP, key persons from KCRI, TMDA and MSD, Director of NIMR's Research Coordination and Promotion, Regional Medical Officers (Mwanza and Simiyu Region)	Identify and deliberate on solutions to address gaps and challenges that may affect the process to introduce and deliver pediatric praziquantel.
		Seek national opinion and approval of the proposed delivery models for pediatric praziquantel.
		Solicit further guidance on linking the proposed delivery models for pediatric praziquantel into existing health system structures delivery.

**FIGURE 1:**
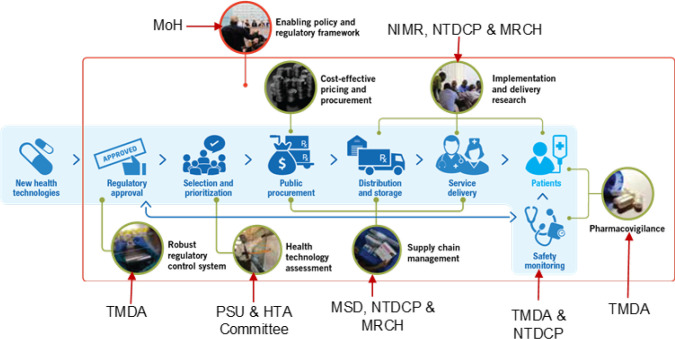
ADP Value Chain for Introduction of New Health Technologies

**Source:** http://adphealth.org/upload/resource/Issue_brief_case_study_of_Tanzania.pdf.

**Abbreviations:** TMDA – Tanzania Medicines and Medical Devices, PSU – Pharmaceutical Services Unit, HTA – Health Technology Assessment, MSD – Medical Stores Department, NTDCP – Neglected Tropical Diseases Control Programme, MRCH – Maternal, Reproductive and Child Health, NIMR – National Institute for Medical Research, MoH – Ministry of Health.

**National level consultative workshop:** A higher level national consultative workshop was held to deliberate and provide solutions to the gaps identified from the technical meeting. The workshop also offered guidance on subsequent activities aimed at streamlining the adoption and introduction of arPZQ in Tanzania. It was chaired by the Chief Medical Officer and attended by Directors and Heads of programs from the Ministry of Health, and representatives from the President's Office Regional Administration and Local Government (Figure 1). Additional participants included WHO/TDR, UNDP, Regional Health Management Teams of Mwanza, Simiyu and Kigoma representing regions with moderate to high prevalence of schistosomiasis, and research and academic institutions including NIMR and Catholic University of health and Allied Sciences (CUHAS). The workshop addressed various gaps such as registration of arPZQ when approved by the WHO, inclusion of arPZQ in the National Essential Medicines List of Tanzania (NEMLT), the appropriate age bundle for children under 5 years (≤5, and potential delivery models to be piloted once the medicine becomes available. Output from this workshop informed the next steps, which expanded the engagement scope from regional health management teams (authorities) to the community level.

**Sub-national level Consultative meetings:** This involved holding engagement meetings with Regional Health Management Teams (RHMTs) and Council Health Management Teams (CHMTs) (Table 1). Regions were selected based on existing epidemiological data from the NTDCP, prioritising those with high to moderate schistosomiasis prevalence., Within each region, councils were further selected according to their documented disease burden. Kigoma and Simiyu regions were selected as representatives of moderate and high schistosomiasis prevalence settings, respectively. Subsequently, Kigoma and Itilima District Councils were selected from these regions for further engagement.

According to the Tanzanian health system structure, Region Health Management Teams (RHMTs) are responsible for overseeing the delivery of health services within their respective region. Their functions include facilitating capacity development, interpretation of policies and guidelines, supporting quality improvement initiatives, and conducting both clinical and managerial supervision. Council Health Management Teams (CHMTs), on the other hand are mandated to plan, implement and coordinate the delivery of healthcare services at the primary healthcare (council) level. Operationally, both RHMTs and CHMTs are responsible for social mobilization, health education and promotion during mass drug administration campaigns. Based on these responsibilities, separate consultative meetings were held with each team in every selected region.

During all meetings, the project team introduced key topics by presenting the objectives of the project, the burden of paediatric schistosomiasis and providing updates on the upcoming arPZQ treatment. Participants were given the opportunity to ask questions, seek clarification, and contribute to open discussion. Thereafter, attendees were divided into two breakout groups for in-depth discussion, followed by plenary sessions to consolidate insights and recommendations.

**Community engagement:** From each of the two selected district councils, two wards (sub-districts) were purposively selected to represent wards with moderate to high prevalence of schistosomiasis. From each ward, 1 community engagement meeting was held thus making a total of 4 community engagement meetings. In Kigoma District Council, meetings were held in Mkongora and Simbo wards, while in Itilima District Council, they were held in Zagayu and Budalabujiga wards. The objectives of community engagement were two-fold: involve the community in the identification of potential challenges which may affect the delivery and uptake of arPZQ, and to gather local insights on the proposed delivery models and suggest workable strategy for promoting the uptake of arPZQ.

Ward leaders in collaboration with village leaders identified key community members capable of meaningfully contributing to discussions and offering solutions to anticipated challenges. Furthermore, identified participants had the potential of leading the efforts of providing information to the community about paediatric schistosomiasis and the imminent availability of arPZQ. In each ward, 34 people participated in the meetings. Their distribution was as follows, 10 to 12 members of Ward Development Committee (WDC), 2 village health committee members, 2 community health workers, 2 health care providers, 2 ward and 2 village leaders, 2 primary school teachers, 4 religious leaders, 4 influential people in the community as determined by ward and village executive officers, 1 NTD ward focal person, and 1 Council NTD coordinator. Each session began with an introduction of the project, its objectives, rationale for community engagement, and the expected deliverables. This was followed by a brief presentation of the status of paediatric schistosomiasis (based on the available epidemiological data), and ongoing global and national control efforts. This was done to raise participants’ awareness on the burden and consequences of paediatric schistosomiasis and set the ground for discussion. Participants had the opportunity to ask questions, provide opinions or to contribute to what has been presented to them. Thereafter, participants were divided into groups of 17 people and requested to identify challenges with the potential to affect the uptake of paediatric praziquantel and propose solutions to the challenges.

### Data Analysis

Information obtained during community engagement and consultative meetings through both group and panel discussions was analyzed using the Thematic Content Analysis approach. The process was iterative, with analysis conducted continuously as the project team progressed through different levels of engagements and consultations. The analysis followed the principles of grounded theory, employing multiple coding to ensure that coding categories accurately reflected the content of the data. Commonalities and differences within the data were systematically analyzed in order to identify response patterns across participants’ diverse experiences.

### Ethical Consideration

The study protocol was approved by the Lake Zone Institutional Review Board on behalf of the National Health Research Ethics Committee with approval Ref. No. MR/53/100/684. Additionally, permission to conduct the study was sought from the President's Office Regional Administration and Local Government. Verbal informed consent was obtained from participants. Information about the study, and contacts of whom to contact in case of questions or concerns during and after study participation were displayed in the consent forms.

## RESULTS

Overall, participants at all levels insisted on the need for integrated service delivery as it would increase access to and delivery of the intervention. Participants believed that treating infected children will have a positive impact on children's growth as well as protecting their siblings and other neighbouring children.

### Identification of systemic barriers to the introduction and delivery of arPZQ

The engagement of Institutions indicated in Figure 1, identified issues which required timely actions. These included i) registration of arPZQ, ii) availability of arPZQ dossier to facilitate the regulatory processes, and development of tools to guide drug dispensing, iii) inclusion of arPZQ in the National Essential Medicine List of Tanzania (NEMLT), iv) financing procurement of arPZQ, v) quantification of arPZQ as no robust data is available on the national burden of paediatric schistosomiasis in the country, vi) inadequate knowledge on the treatment of paediatric schistosomiasis among health workers at PHC facilities and communities, and vii) lack of joint planning meetings between NTDCP and MRNCH which would fast track the integration of NTD in clinical services for children aged ≤5 year old.

### National level validation and commitments

The national higher level engagement workshop was chaired by the Chief Medical Officer of Tanzania, and in the presence of the key directors from the Ministry of Health, the President's Office, Regional Administration and Local Government. The workshop validated the proposed strategies for delivery of arPZQ, and the strategies for promoting uptake of arPZQ. Furthermore, Pharmaceutical Services Unit addressed gaps related to registration and inclusion of arPZQ in the NEMLT. In this regard, the unit committed to fast tract inclusion of arPZQ in the NEMLT and standard treatment guidelines (STGs) upon registration by the TMDA. This is because the parent drug, praziquantel is already included in NEMLT, and hence the meeting was informed it will be easy to include a re-worked arPZQ formulation in the key national guideline documents for medicines.

*“It should not be a problem to include the drug into NEMLT and STG, as praziquantel is already in the list. It will be easy to include the paediatric formulation in the NEMLT* (Participant from PSU)”

The deliberation about delivery models provided a better understanding of how best the strategies or delivery approaches should be structured. The decision makers recognized all proposed delivery models as “integrated delivery models”. About the integration of the delivery of arPZQ in the child health and growth monitoring services, it was agreed that this strategy should be implemented through Maternal and Child Health Services (MCH) and Outpatient Department (OPD). This is because when a child reaches 3 years, it is rare to see a parent taking the children for growth monitoring or any child health services at the MCH clinic. Therefore, most children aged 3 to 5 years will easily be accessed through OPD. One of the assertions from the workshop participants commented as follows;

*“It is possible and convenient to deliver arPZQ through MCH services where children aged 2 to 3 years can be reached easily. However, children aged 4 to 5 years can be reached in pre-school settings or community-based MDA. In terms of minimum age, the MRCH department advise that, arPZQ should be administered to children aged 2 to 5 years (Participant from Maternal, Reproductive and Child Health)”.*

Regarding MDA, participants confirmed that targeting children aged 3 to 5 years old through MDA is possible, however there will be need to conduct a thorough awareness campaign to address knowledge, attitude, and myth among caretakers and parents. MDA will efficiently compliment the proposed test and treat approach through the MCH clinic and OPD.

### Health system and community readiness to promote the uptake of arPZQ

Community, CHMT and RHMT members welcomed the opportunity to have a new intervention targeting children aged ≤5 year who are infected with schistosomiasis.

*“We are happy that there is hope for schistosomiasis control in children under the age of 5 years. Now to overcome misconceptions and myths about drug intervention, it is important that we start raising community awareness on schistosomiasis control to prepare the communities to receive the drug* (Itilima CHMT Member)”.

There was a consensus on the use of a communityowned innovative strategy to create awareness on paediatric schistosomiasis and prepare the community to take up the drug. In this aspect, participants proposed to use individuals within the community to serve as advocate for anti-schistosomiasis campaign. The resource persons who can be used as Community Anti-Schistosomiasis Advocates (CASA) included local political and community leaders, influential elders, traditional health practitioners, community health officers and healthcare workers. However, appropriate criteria which will guide the objective recruitment of CASA need to be developed. These resource persons can be trained to provide health education on paediatric schistosomiasis, the burden of the disease among children aged ≤5 year, consequences if not treated, control and prevention strategies. They will further receive training on how to engage the communities in raising awareness on paediatric schistosomiasis treatment and prevention, and the importance of Water, Sanitation, and Hygiene (WASH) in preventing transmission of schistosomiasis. Some assertions from group discussions included;

*“In each village there are 1 – 2 community health workers who can mobilize parents and guardians of pre-school children to seek paediatric schistosomiasis services when available in health facility, community and during special campaigns such as Vitamin A supplementation* (Kigoma CHMT Member)”.

*“Having local resource persons to promote prevention and control of paediatric schistosomiasis will help to enhance community involvement, participation and ownership, ultimately increase demand and uptake of the drug* (Simiyu RHMT Member)”

Participants emphasized building strong relationships, effective communication and interactions between the beneficiaries of the interventions, community members, CHMTs and RHMTs to influence uptake of paediatric praziquantel once available for use.

At the community level, participants recommended that before arPZQ is introduced for use, the following should be done; a) Use the most influential groups in the community and local resource persons to develop appropriate IEC/BCC materials, b) Continuous provision of health education to equip the community with knowledge and skills to prevent and control paediatric schistosomiasis, c) Organize events or leverage on the existing platforms such as village meetings to raise community awareness on the burden and consequences of paediatric schistosomiasis

To ensure adherence to protocol for delivering health education related to prevention and control of paediatric schistosomiasis. CHMT and RHMT members expressed the needs for capacity strengthening for provision of supportive supervision.

*“We should not forget the needs for supportive supervision of health care providers and community health workers and other key people involved in creation of community awareness and provision of health education on paediatric schistosomiasis. I mean capacities of RHMTs and CHMTs for provision of supportive supervision during the delivery of schistosomiasis control and treatment interventions should be strengthened, this is a new intervention focusing in a group which has never been covered in the existing efforts to control the disease* (Simiyu RHMT Member)”

### Proposed delivery strategies for arPZQ

The engagement of multi-stakeholders from national to community level brought consensus on the appropriate delivery strategies for arPZQ. With some input for improvements noted at each level of engagement, all stakeholders supported the integrated approach for delivery of arPZQ. Using the existing platforms for delivering various health and medical interventions for children aged ≤5 year, the following delivery strategies were deemed appropriate to the context of the Tanzanian health system; a) Integrated delivery of arPZQ with the national vitamin A supplementation and deworming programme, b) Integration of delivery of arPZQ into child health and growth monitoring services and c) delivery of arPZQ through community mass drug administration (Community based pre-SAC MDA). For the strategy which integrates the delivery of arPZQ into child healthcare and growth monitoring services, the WHO recommended test and treat approach will be adopted. It was further emphasized that prior to deployment of arPZQ, guidelines and strategies to guide the implementation should be developed and disseminated widely to enable the implementers to do the right thing.

*“There is a great need for strengthening the capacity of RHMTs and CHMTs to provide supportive supervision to ensure that the drug is delivered in accordance with the protocols for each strategy* (Kigoma RHMT Member)”

Recognizing the critical role of WASH in transmission of schistosomiasis infection, RHMT, CHMT and community members proposed that the delivery of arPZQ should go hand in hand with WASH interventions. It was proposed that, regions and councils develop strategies to enforce bylaws requirine each household to construct acceptable toilet, and to adhere to recommended sanitation and hygiene practices.

## DISCUSSION

The combination of top-down, and bottom-up approaches in stakeholders’ engagement effectively linked community perspectives with national level decision making and vice-versa. There were strong alignments of views across all levels. Stakeholders agreed that using existing platforms to deliver arPZQ may reduce the delivery cost while ensuring high treatment coverage. The proposed strategy to use CASA in raising awareness about the availability of arPZQ and the providing health education about schistosomiasis and its determinants was well received by all stakeholders. The adoption of an integrated approach to delivery of arPZQ, using the community as the driver of the programme through CASA resembles the community directed treatment with ivermectin (CDTI) strategy implemented in Cameroon.^16^ Thus, understanding what worked and what did not work with CDTI may inform the best way to implement the CASA strategy and the delivery of arPZQ through integrated strategies.^17,18^ The lessons from CDTI are particularly relevant for conducting community MDA targeting children aged 2 to 5 years.

The test and treat approach will be used at health facilities which provide MCH services. As recommended by the higher-level workshop participants, the test and treat approach will further be extended to OPD to ensure that children who qualify to get the intervention do not miss treatment. According to the guidance of decision makers in the health sector, the arPZQ will be dispensed to children aged 24 to 59 months after being diagnosed positive of schistosomiasis. Stakeholders, including the RHMT and CHMT, emphasized the need to strengthen the capacity of healthcare workers at the primary healthcare (PHC) facilities to improve their competence in diagnosing and treating paediatric schistosomiasis. One of the pressing challenges faced by PHC facilities is limited number of Laboratory technologists skilled in microscopical diagnosis using Kato-Katza and Urine filtration methods. This gap could be addressed either by training existing staff or by providing rapid diagnostic tests such as Point of Care Circulating Cathodic Antigens (POC-CCA) to facilities selected for the pilot delivery of arPZQ.

For integrated delivery of arPZQ with vitamin A supplementation and deworming programs, which is implemented twice a year, health workers involved at both facility and community levels must be trained on how to deliver appropriate dosage to children aged 24 to 59 months. Unlike MDA for School Aged Children, where a dose pole is used as a height to weight dosing, the delivery of arPZQ is based on the child's weight, thus during Pre-SAC MDA, weighing scales must be made available to avoid under or overdosing Frontline health workers or community drug distributors will have to be trained, and provided with job aids to ensure proper delivery. On the other hand, CASA will be used to mobilize parents and guardians to bring their children to designated drug distribution points at the health facilities or in the community. Similar processes will apply if a community based MDA model for children aged 2 to 5 years is implemented.

The engagement of the community level brought excitement when they learned of the imminent availability of arPZQ for the treatment of schistosomiasis in children ≤5 years. This is because some of them have seen children with schistosomiasis symptoms as they live in areas with high prevalence of this NTD., The community's suggestion to deliver arPZQ along with WASH interventions corroborates with the views of Wami et al,^19^ who demonstrated that poor provision of sanitation, supply of clean and safe water and limited health education impact negatively on prevalence and intensity of schistosomiasis. Of uttermost importance was the demand by the communities to drive the health education and awareness campaign themselves. This is by capacitating the communities to be the change agents and advocates against schistosomiasis. This is what gave birth to the CASA strategy, which received broad support form sub-national and national stakeholders.

Oveall, engagement across national, regional, council and community levels revealed strong readinessand acceptance for the introduction of arPZQ in Tanzania. Consensus was reached that arPZQ delivery should adopt an integrated approach, combining routine healthcare services via a test and treat model with community based Pre-SAC MDA, either independently or integrated with Vitamin A supplementation and deworming programs.

## CONCLUSION

The adoption of an Implementation Research (IR) strategy to strengthen the health system for the introduction and delivery of arPZQ in Tanzania has produced valuable insights. Guided by the 3 key steps of IR; pre-intervention assessment, project implementation with continuous monitoring, and evaluation of the project, the initial phase of the project has yielded actionable outputs. On pre-intervention, several milestones were achieved. First, as a country, the stakeholders were able to identify pertinent gaps, some of which needed immediate solutions, while some needed time to be addressed. On this, gaps related to the inclusion of arPZQ in the NEMLT and the STG got some answers, all that remains is to implement solutions when the drug is registered in Tanzania. Second, delivery models which are contextually relevant were identified and validated at the national level, paving way for development of strategies and protocol for the pilot deployment of arPZQ in Tanzania. Third, community engagement provided addition inputs and suggestions on how best arPZQ can be delivered in the country, culminating in a national consensus on delivery models, strategies, and measures to ensure community ownership and support of the intervention.

These achievements mark the conclusion of the pre-intervention phase of the project. Work is underway to implement the pilot deployment of arPZQ using the proposed delivery models discussed in this article. This will be done with continuous monitoring of the implementation and efficacy of the strategies as well as the impact of the arPZQ on the well-being of children ≤5year old in the selected study sites.
